# HIV-1 Gag Blocks Selenite-Induced Stress Granule Assembly by Altering the mRNA Cap-Binding Complex

**DOI:** 10.1128/mBio.00329-16

**Published:** 2016-03-29

**Authors:** Alessandro Cinti, Valerie Le Sage, Marwan Ghanem, Andrew J. Mouland

**Affiliations:** a HIV-1 RNA Trafficking Laboratory, Lady Davis Institute at the Jewish General Hospital, Montréal, Québec, Canada; b Department of Medicine, McGill University, Montréal, Québec, Canada; c Department of Microbiology and Immunology, McGill University, Montréal, Québec, Canada

## Abstract

Stress granules (SGs) are dynamic accumulations of stalled preinitiation complexes and translational machinery that assemble under stressful conditions. Sodium selenite (Se) induces the assembly of noncanonical type II SGs that differ in morphology, composition, and mechanism of assembly from canonical SGs. Se inhibits translation initiation by altering the cap-binding activity of eukaryotic translation initiation factor 4E (eIF4E)-binding protein 1 (4EBP1). In this work, we show that human immunodeficiency virus type 1 (HIV-1) Gag is able to block the assembly of type II noncanonical SGs to facilitate continued Gag protein synthesis. We demonstrate that expression of Gag reduces the amount of hypophosphorylated 4EBP1 associated with the 5′ cap potentially through an interaction with its target, eIF4E. These results suggest that the assembly of SGs is an important host antiviral defense that HIV-1 has evolved for inhibition through several distinct mechanisms.

## IMPORTANCE

The antiviral stress response is an important host defense that many viruses, including HIV-1, have evolved to evade. Selenite induces a block in translation and leads to stress granule assembly through the sequestration of eIF4E by binding hypophosphorylated 4EBP1. In this work, we demonstrate that in the face of selenite-induced stress, HIV-1 is able to maintain Gag mRNA translation and to elicit a blockade to selenite-induced stress granule assembly by altering the amount of hypophosphorylated 4EBP1 on the 5′ cap.

## INTRODUCTION

The host translational machinery is regulated by environmental stresses, which trigger multiple signaling pathways leading to either cell survival or cell death. Cellular stress initiates the assembly of cytoplasmic aggregates called stress granules (SGs) that consist of dynamic accumulations of stalled translation preinitiation complexes. Nucleation of several canonical factors such as Ras GTPase-activating protein-binding protein 1 (G3BP1) and T-cell intracellular antigen (TIA-1) and its receptor, TIAR (1–3), is required to assemble SGs.

Human immunodeficiency virus type 1 (HIV-1) is the etiological agent of acquired immunodeficiency syndrome (AIDS). The structural polyprotein pr55^Gag^ (referred to here as Gag) assembles at the plasma membrane to form HIV-1 particles. Upon budding and release, the virion becomes infectious only after proper processing of Gag into the mature proteins: matrix (MA), capsid (p24^CA^), nucleocapsid (NC), and p6. The subversion of host machineries is an essential part of the virus replicative process, and, similarly to many other viruses, HIV-1 has evolved to corrupt components of SGs to promote viral replication by blunting or eliminating antiviral host defenses (4). HIV-1 Gag, specifically, the amino-terminal domain of p24^CA^, mediates the disassembly of preexisting SGs in part due to an interaction with G3BP1 (5). Moreover, when cells are exposed to oxidative stress (by arsenic [Ars]), p24^CA^ elicits this blockade to SG assembly through a direct interaction with the translation factor, eukaryotic elongation factor 2 (eEF2) (5, 6).

Selenium is an essential micronutrient that is incorporated into selenoproteins and has antioxidant properties that protect against cancer (7). Previous studies indicated that chronic selenium deficiency appears linked to increased viral pathogenicity and the evolution of more-virulent RNA viruses (8). Reported outcomes of different selenium intervention trials, although somewhat inconsistent, suggest that supplementation may delay the progress to AIDS, slow the depletion of CD4^+^ T cells, and reduce morbidity (9–13). Sodium selenite (Se) is the commercially available version of selenium. In human osteosarcoma U2OS cells, Se causes mRNA translational repression followed by assembly of noncanonical type II SGs, which differ in size, localization, composition, and mechanism of assembly from those induced by Ars (14). Cap-dependent translation requires the binding of eukaryotic initiation factor 4E (eIF4E) to the 7-methylguanosine (m^7^G) cap structure, as part of the eIF4F complex, consisting of eIF4E, eIF4G, and eIF4A. Mammalian target of rapamycin complex 1 (mTORC1) finely tunes translation initiation by phosphorylating its substrate, eIF4E-binding protein 1 (4EBP1). In this scenario, phospho-4EBP1 does not associate with eIF4E to allow translation. However, Se inactivation of mammalian target of rapamycin (mTOR) kinase activity leads to hypophosphorylation of 4EBP1 and a concomitant increase in 4EBP1:eIF4E binding on the 5′ cap (14, 15), which inhibits assembly of the eIF4F complex to reduce mRNA translation initiation (16). The block to translation ultimately results in the assembly of SGs.

We explored the question of whether HIV-1 was capable of blocking Se-induced SG assembly and how Se stress impacted HIV-1 mRNA translation and replication. We found that HIV-1 blocks Se-induced SG assembly to facilitate continued viral mRNA translation. Furthermore, we show that the HIV-1 structural protein, Gag, elicits the blockade and does so by using a novel mechanism of inhibition. Gag immunoprecipitates with the 5′ cap and interacts with eIF4E to reduce the amount of hypophosphorylated 4EBP1 associated with the 5′ cap. Importantly, Se was found to have a detrimental effect on Gag processing and infectivity of released HIV-1 particles.

## RESULTS

### HIV-1 blocks the assembly of Se-induced SGs.

**FIG 1:**
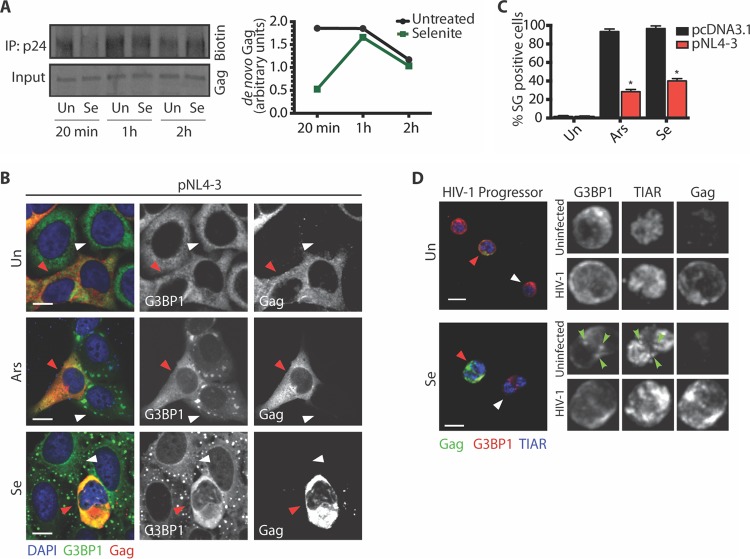
HIV-1 blocks Se-induced SG assembly. (A) U2OS cells were transfected with pNL4-3 and treated without (Un) or with 1 mM Se in medium containing AHA. At the indicated times, cell lysates were collected and click chemistry was performed followed by immunoprecipitation (IP) with rabbit anti-p24 antibody. Densitometry quantification of *de novo* synthesized, biotinylated Gag was performed by ImageJ analysis. Values presented in the graph are normalized against the total amount of Gag in the cell lysate. (B) U2OS GFP-G3BP1-expressing cells were transfected with pNL4-3 and left untreated (un) or treated with 500 µM Ars for 45 min or 1 mM Se for 2 h. Cells were stained for DAPI (blue) and Gag (red). White arrowheads indicate nontransfected cells, while red arrowheads show HIV-1-expressing cells. The gain of Gag under conditions of Ars treatment was reduced to better visualize the absence of SGs. Scale bars are 10 µm. (C) Quantification of U2OS cells transfected with pcDNA3.1 (black) or pNL4-3 (red) containing SGs from the experiment represented in panel B. Error bars represent standard deviations of results from three independent experiments, with 150 cells counted per treatment. Asterisks represent statistically significant differences between mock treatment and HIV-1-expressing cells (two-way analysis of variance [ANOVA]; *P* < 0.001). (D) PBMCs from a healthy untreated HIV^+^ progressor were left untreated (un) or exposed to 1 mM Se for 2 h. Cells were stained for TIAR (blue), G3BP1 (green), and Gag (red). White arrowheads indicate noninfected cells, while red arrowheads indicate HIV-1-infected cells, identified by Gag immunoreactivity. In the inset, green arrowheads indicate SGs with colocalization of G3BP1 and TIAR. Scale bars are 10 µm.

Se induces a translational blockade that causes accumulation of SGs (14), with the most robust assembly being evident as early as 2 h post-treatment (see Fig. S1 in the supplemental material) (14). To investigate the effect of Se on Gag synthesis, HIV-1-transfected U2OS cells were treated without or with Se and newly translated proteins were labeled with AHA (l-azidohomoalanine), a methionine analog, and visualized using click chemistry. Compared to the results seen with untreated controls, there was a reduction in Gag expression after 20 min in the presence of Se, which increased to similar levels at 1 and 2 h posttreatment (Fig. 1A). These data suggest that HIV-1 was able to overcome the initial translational block imposed by Se and restore protein synthesis.

HIV-1 imposes a blockade on the assembly of SGs by Ars or pateamine A (PatA) or in cells overexpressing G3BP1/TIAR (5, 6). Se induces the assembly of noncanonical type II SGs that contain core SG markers (G3BP1, TIA-1, and TIAR) but lack eIF3 (see Fig. S2A and B in the supplemental material) via a mechanism that is dependent upon the production of reactive oxygen species (ROS) (see Fig. S2C) (14). To determine the ability of HIV-1 to block the assembly of Se-induced SGs, U2OS cells stably expressing green fluorescent protein-G3BP1 (GFP-G3BP1) (17) were subjected to mock (pcDNA3.1) or HIV-1 (pNL4-3) transfection and subsequently stressed with Se or Ars. In Se-treated cells transfected with pcDNA3.1, SGs were observed in 95% of cells (Fig. 1C), while only 40% of HIV-1-expressing cells possessed SGs (Fig. 1B, red arrowhead, and C). This reduction in the number of HIV-1-expressing cells containing SGs in the presence of Se was comparable to that observed upon treatment with Ars (28%) (Fig. 1B, red arrowhead, and C). To examine the assembly of Se-induced SGs in HIV-1 patient CD4^+^ T cells, peripheral blood mononuclear cells (PBMCs) from a healthy untreated progressor were stressed with carrier alone or Se. Upon treatment with Se, SGs were not visible in HIV-1-infected PBMCs (Fig. 1D, red arrowhead), while those cells that did not stain positively for Gag were able to assemble SGs upon treatment, as evidenced by the colocalization of G3BP1 and TIAR (Fig. 1D, white arrowhead). Similar results were obtained with Se-treated NL4-3-infected Jurkat T cells compared to mock-infected cells (see Fig. S3). These data demonstrate that HIV-1 is able to block the assembly of Se-induced noncanonical type II SGs.

### Gag blocks the assembly of Se-induced SGs.

**FIG 2:**
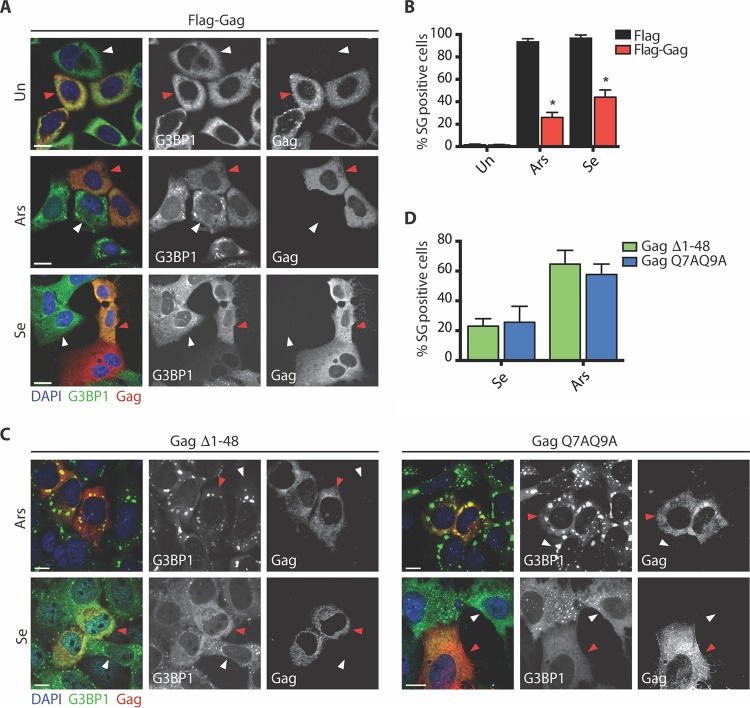
Gag specifically blocks Se-induced SG assembly. (A) U2OS GFP-G3BP1-expressing cells transfected with Flag (not shown) or Flag-Gag were left untreated (un) or incubated with 500 µM Ars for 45 min or 1 mM Se for 2 h. Cells were stained for DAPI (blue) and Gag (red). White arrowheads indicate nontransfected cells, while red arrowheads show Flag-Gag-expressing cells. Scale bars are 10 µm. (B) Quantification of U2OS GFP-G3BP1-expressing cells transfected with Flag (black) or Flag-Gag (red) from the experiment described for panel A. Error bars represent standard deviations of the results of three independent experiments, with 150 cells counted per treatment. Asterisks represent statistically significant differences between Flag-expressing and Flag-Gag-expressing cells (two-way ANOVA; *P* < 0.001). (C) U2OS GFP-G3BP1-expressing cells transfected with either Δ1-48 or Q7AQ9A Gag mutants were incubated with 500 µM Ars for 45 min or 1 mM Se for 2 h. Cells were stained for DAPI (blue) and Gag (red). White arrowheads indicate nontransfected cells, while red arrowheads show mutant Gag-expressing cells. Scale bars are 10 µm. (D) Quantification of U2OS GFP-G3BP1-expressing cells transfected with Gag Δ1-48 (green) or Gag Q7AQ9A (blue) containing SGs from the experiment described for panel A. Error bars represent standard errors of the means of the results of three independent experiments, with 100 cells counted per treatment.

The HIV-1 structural protein Gag has been previously demonstrated to impose a blockade on the assembly of SGs induced by Ars (5). To determine the ability of HIV-1 to inhibit the assembly of Se-induced SGs, U2OS cells stably expressing GFP-G3BP1 were transfected with Flag or Flag-Gag and exposed to Ars or Se. In 95% of cells transfected with Flag alone, SGs were apparent under Ars- and Se-treated conditions (Fig. 2B). As shown in Fig. 2A (red arrowhead), those untreated cells transfected with Flag-Gag had no visible SGs, while 26% and 44% had SGs in the presence of Ars and Se, respectively (Fig. 2A, red arrowhead, and B). The N-terminal domain of HIV-1 p24^CA^ interacts with eEF2 to block Ars-induced SG assembly (5). To determine if the blockade to SG assembly by Ars and Se is mediated by this domain in Gag, U2OS cells stably expressing GFP-G3BP1 were transfected with one of two Gag mutants, Δ1-48 or Q7AQ9A, and incubated with Ars or Se. As expected, 65% or 58% of cells transfected with either the Δ1-48 or Q7AQ9A Gag mutant, respectively, displayed SGs upon treatment with Ars (Fig. 2C and D). Interestingly, each of these Gag mutants was still able to block the assembly of SGs in the presence of Se, with only 23% for the Δ1-48 mutant and 25% for the Q7AQ9A mutant having visible SGs (Fig. 2C and D) indicating that the imposed blockades of Ars- and Se-mediated SG assembly by Gag are not mechanistically linked. These data indicate that HIV-1 Gag is sufficient for the HIV-1-mediated inhibition of Se-induced SG assembly.

### HIV-1 does not affect the phosphorylation status of S6K or 4EBP1 in the presence of Se.

**FIG 3:**
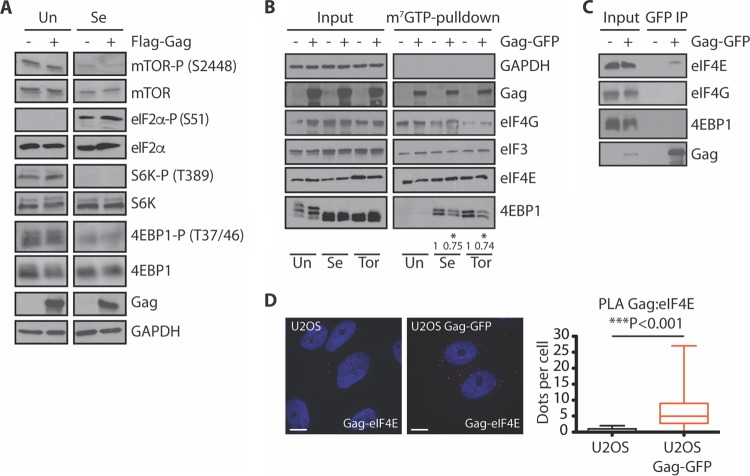
Gag expression reduces the amount of hypophosphorylated 4EBP1 associated with the 5′ cap, without changing the activation status of mTOR. (A) U2OS cells were transfected with Flag or Flag-Gag for 18 h before treatment without or with 500 µM Ars for 45 min or 1 mM Se for 2 h. Cell lysates were subjected to SDS-PAGE, immunoblotted, and probed with the indicated antibodies. Data shown are representative of the results of three independent experiments. (B) U2OS cells expressing GFP or Gag-GFP were treated with Se (1 mM for 2 h) or the mTOR inhibitor Tor (250 mM for 1 h). Cell lysates were collected and subjected to m^7^GTP agarose pulldown. Cap-associated proteins were processed for Western blotting and probed for GAPDH, (p24) eIF3, eIF4E, and 4EBP1. Densitometry quantification of 4EBP1 associated with the 5′ cap was performed by ImageJ analysis. Fold changes in the amount of protein pulled down are indicated below each lane. Each value was normalized against the total amount of protein input; for each condition, the value for GFP alone was arbitrarily set at 1. Asterisks represent statistically significant differences between GFP-expressing and Gag-GFP-expressing cells (Student’s *t* test; *P* < 0.001). (C) Cell lysates from U2OS cells expressing GFP or Gag-GFP were immunoprecipitated with anti-GFP MAb magnetic beads. The immunoprecipitate was analyzed by Western blotting with anti-eIF4E, anti-eIF4G, anti-4EBP1, and anti-p24 antibodies, as indicated. (D) U2OS GFP-expressing or Gag-GFP-expressing cells were fixed, permeabilized, and incubated with mouse anti-p24 and rabbit anti-eIF4E. Coverslips were subsequently incubated with anti-mouse and anti-rabbit PLA probes. Each red dot corresponds to a single event of interaction between Gag and eIF4E. Nuclei were stained with DAPI (blue). Images shown are representative of 70 cells analyzed in 2 independent experiments. The graph indicates the number of dots per cell. Asterisks represent statistically significant differences between GFP-expressing and Gag-GFP-expressing U2OS cells (Student’s *t* test; *P* < 0.001).

mTOR is a serine/threonine kinase that plays important roles in cell growth and proliferation. As a component of the mTORC1 complexes (18), mTOR regulates cap-dependent mRNA translation through the phosphorylation of ribosomal S6 kinase (S6K) and 4EBP1 (19). Mechanistically, Se induces the assembly of SGs by inhibiting mTOR activity through the production of ROS, which results in dephosphorylation of 4EBP1 and increased eIF4E:4EBP1 binding to block translation (14). Additionally, SG assembly is driven by eIF2α phosphorylation resulting from endoplasmic reticulum stress triggered by Se (20, 21). To elucidate the mechanism of HIV-1 Gag SG blockade in response to Se stress, we first examined the activation status of the mTOR pathway. U2OS cells were transfected with either Flag or Flag-Gag and subsequently stressed with 1 mM Se. Compared to untreated cells, Se-exposed cells had reduced phosphorylation levels of mTOR, S6K, and 4EBP1, indicating that the mTOR pathway was repressed, while Se treatment increased phospho-eIF2α (Fig. 3A) (14). Under conditions of Se stress, the phosphorylation status of mTOR, S6K, 4EBP1, and eIF2α was unaltered by expression of Flag-Gag compared to expression of Flag alone. The blockade of Se-induced SG assembly by HIV-1 Gag does not appear to be regulated by eIF2α phosphorylation or via the mTOR pathway but may be regulated at a point downstream of the mTOR node.

### HIV-1 Gag reduces the amount of hypophosphorylated 4EBP1 associated with the 5′ cap.

Se-induced SG assembly is dependent on increased association of 4EBP1 with the 5′ cap, which inhibits translation initiation (14). In a further attempt to identify the mechanism by which Gag inhibits Se-induced SG assembly, we investigated the association of 4EBP1 with the 5′ cap in Gag-expressing cells. U2OS cells expressing GFP or Gag-GFP were treated with Se or Torin (Tor). Tor is an ATP-competitive mTOR inhibitor that blocks 4EBP1 phosphorylation and consequently increases 4EBP1 binding to eIF4E as well as association with the 5′ cap (Fig. 3B) (22). Tor treatment was included as a positive control. We performed a pulldown assay using agarose beads conjugated with a m^7^GTP cap analog and observed that HIV-1 Gag precipitated in untreated and Se- and Tor-treated cells to similar extents (Fig. 3B), which were independent of RNA (see Fig. S4B in the supplemental material). Treatment with Se and Tor resulted in hypophosphorylated 4EBP1, and, as expected, the amount of 4EBP1 associated with the 5′ cap increased under these stress conditions (Fig. 3B) (14). In Gag-expressing cells, a clear and consistent decrease (25% ± 4; *n* = 4) in the level of hypophosphorylated 4EBP1 was observed in both Se- and Tor-treated cells, whereas there was no apparent difference in the levels of association of 4EBP1 with the 5′ cap in untreated cells (Fig. 3B). These data suggest that association of Gag reduced the amount of hypophosphorylated 4EBP1 on the 5′ cap to allow continued translation of Gag. Moreover, the decreased association of hypophosphorylated 4EBP1 was specific as there was no difference in eIF4E binding to the 5′ cap in the presence of Gag (Fig. 3B).

Cap-dependent translation is inhibited through the competitive binding of hypophosphorylated 4EBP1 to eIF4E on the 5′ cap, which disrupts the eIF4F complex (23). We explored the potential for Gag to disrupt the interaction of eIF4E and 4EBP1 by examining the binding of Gag to each of these host proteins. As shown in Fig. 3C, using anti-GFP beads to immunoprecipitate Gag-GFP, we demonstrated that eIF4E was specifically pulled down with Gag-GFP but not with GFP alone, whereas neither 4EBP1 nor eIF4G coimmunoprecipitated with Gag-GFP. The proximity ligand assay (PLA) is a highly specific and sensitive method that produces distinct, countable spots representing an endogenous protein-protein interaction at as little as 40 nm (24, 25). PLA was used to localize the eIF4E:Gag interaction in unmodified cells at single-molecule resolution. Strong cytoplasmic complex formation was observed with 6.98 ± 0.8 dots per cell in Gag-GFP-expressing cells compared to 0.31 ± 0.08 dots in control cells (Fig. 3D). Taken together, these data suggest that the inhibition of Se-induced SG assembly may be due to a decrease in hypophosphorylated 4EBP1 association with the 5′ cap as it is potentially displaced by Gag, which was shown to associate with eIF4E.

### Se treatment reduces HIV-1 infectivity.

**FIG 4:**
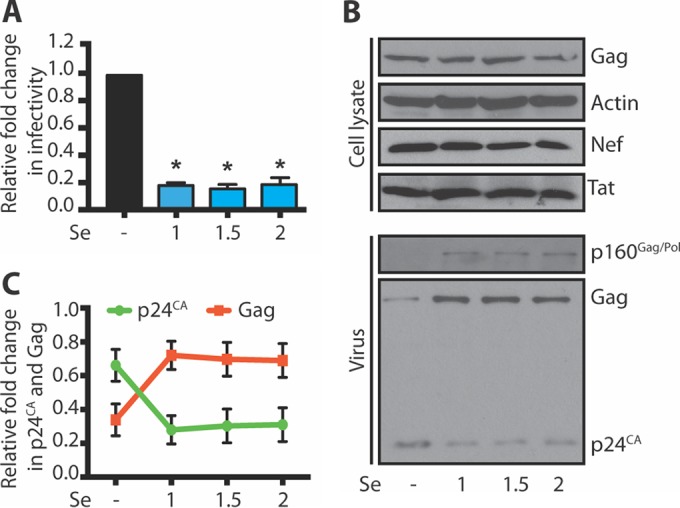
Se decreases the infectivity of HIV-1 particles. (A) U2OS cells were transfected with pNL4-3 and stressed without or with 1, 1.5, or 2 mM Se for 2 h. Virus was collected upon filtration and ultracentrifugation of the supernatant. Infection of TZM-bl cells was performed, cells were lysed, and luciferase activity was measured (bars). Error bars represent standard deviations of the results of three independent experiments. Asterisks represent statistically significant differences between untreated and Se-treated conditions (one-way ANOVA; *P* < 0.01). (B) Cell lysates and virus from the experiment represented in panel A were subjected to Western blotting using anti-p24, Nef, Tat, and β-actin antibodies. (C) Densitometry quantification of the p24^CA^ and Gag levels in virus shown in panel A was performed by ImageJ analysis. The values represent the intensities of p24^CA^ and Gag in purified virus in each lane and are expressed as a relative fold change of the total signal for each lane. Error bars represent standard errors of the means of the results of three independent experiments.

Evidence suggests that dietary Se supplementation improves HIV-1 disease outcomes through an unknown mechanism (9–13). To understand the impact of Se on HIV-1, we first examined HIV-1 production in response to Se treatment. U2OS cells were transfected with the HIV-1-expressing pNL4-3 plasmid before stressing the cells with increasing concentrations of Se. As seen in Fig. 4A, a significant decrease in HIV-1 infectivity was observed upon incubation with increasing concentrations of Se. To examine the reduction of HIV-1 particle infectivity, we performed Western blotting on cell lysates and purified virions. Se treatment did not cause a significant difference in cellular Gag, Nef, and Tat expression levels (Fig. 4B), which is consistent with the observed block in SG assembly (Fig. 1 and 2). However, in purified virus particles, we observed a 2.1-fold (*n* = 3) decrease in fully processed p24^CA^ levels with a concomitant increase in the quantity of Gag, even at the lowest concentration of Se, compared to the untreated control (Fig. 4B and C). Taken together, these data indicate that Se treatment has a detrimental effect on HIV-1 infectivity by inhibiting the processing of Gag.

## DISCUSSION

The salient observation made in this paper is that of the ability of HIV-1 to co-opt the host translational machinery necessary for the assembly of Se-induced SGs. The interaction of HIV-1 Gag with eIF4E (Fig. 3), taken together with the decrease in 5′ cap binding of hypophosphorylated 4EBP1 (Fig. 3), suggests that disrupting eIF4E:4EBP1 produces a block in Se-induced SG assembly (Fig. 1 and 2). Although SG assembly is inhibited by HIV-1, we also demonstrated that Se has a detrimental effect on the processing of the full-length Gag polyprotein in released HIV-1 particles, which has a negative impact upon HIV-1 infectivity (26).

Se induces the assembly of bona fide SGs that are morphologically smaller and lack the core SG component eIF3b compared to those SGs induced by Ars (14). Additionally, the kinetics of SG assembly were slower, with robust SGs being observed only after 2 h of Se treatment (see Fig. S1 in the supplemental material) (14). *De novo* synthesis of HIV-1 Gag was reduced upon addition of Se after 20 min but rapidly rebounded to levels similar to those seen under the untreated condition (Fig. 1A). This lag in time between the initial reduction of Gag expression and the much later assembly of SGs may indicate that Se has an immediate effect on mRNA translation that is quickly overcome by the virus. Early effects of Se on mRNA translation or stability may be in effect before SGs are visible by immunofluorescence. It has been previously shown that Se regulates several cellular transcription factors (AP-1, NFκB, p53) (27); however, microarray studies comparing rats fed 20× the nutritional requirement of Se did not result in significant gene expression changes (28, 29).

**FIG 5:**
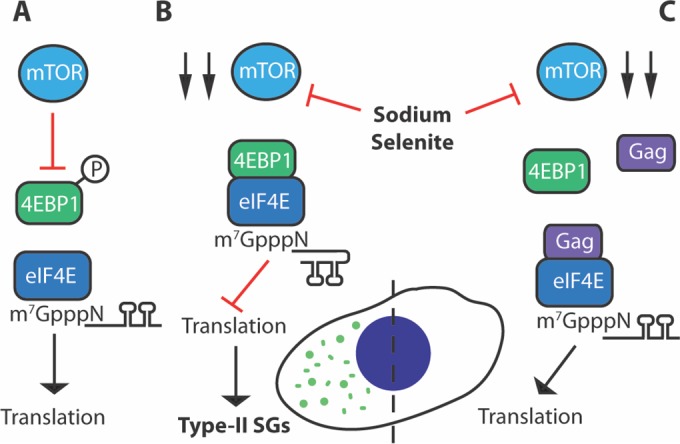
Model for the Gag-mediated blockade of Se-induced SG assembly. (A) In the absence of stress, mTOR is active and stimulates cap-dependent translation through activation of the mTORC1 kinase complex. Phosphorylation of 4EBP1 reduces binding to eIF4E, which allows initiation of mRNA translation. (B) Se triggers a reduction in mTOR activity, thereby increasing binding of hypophosphorylated 4EBP1 to eIF4E on the 5′ cap, which leads to translation inhibition and noncanonical type II SG assembly (green foci). (C) Gag interacts with eIF4E and is found associated with the 5′ cap. The amount of hypophosphorylated 4EBP1 associated with the 5′ cap is reduced in the presence of Gag under conditions of Se and Tor stress to consequently alleviate translation inhibition and promote disassembly of SGs.

Two major cap-binding components, the nuclear cap-binding complex (CBC) and the eIF4F complex, which are predominantly nuclear and cytoplasmic, respectively, are responsible for regulating mammalian translation initiation. CBC is a heterodimer composed of CBP20 and CBP80 (30) and is transported with the mRNA from the nucleus to the cytoplasm (31). The CBC is replaced upon mRNA export by the eIF4F complex to initiate cap-dependent translation, whereas inhibition of cap-dependent translation occurs upon interaction of hypophosphorylated 4EBP1 and eIF4E (32). Se-induced SG assembly is driven by 4EBP1 binding to eIF4E on the 5′ cap (14). Our results show that HIV-1 Gag associated with the 5′ cap potentially through a specific interaction with eIF4E as shown by coimmunoprecipitation and *in situ* PLA (Fig. 3C and D). Regulation of Se-induced SG assembly in HIV-1-expressing cells is not dependent upon the mTOR signaling pathway (Fig. 3A); rather, the presence of HIV-1 Gag affects the association of 4EBP1 with the 5′ cap, without changing its phosphorylation status (Fig. 3A and B). Thus, we propose that HIV-1 Gag interacts with eIF4E to antagonize eIF4E:4EBP1, which alleviates the imposed translational repression to cause SG disassembly (Fig. 5). A similar mechanism of translational regulation was shown in cells infected with the double-stranded DNA virus human cytomegalovirus (HCMV), whereby 4EBP1 is excluded from the mRNA cap-binding complex during infection (33, 34). Although HCMV infection does not induce the assembly of SGs, the virus is able to block SG assembly induced by thapsigargin, a known inducer of endoplasmic reticulum stress (35). Earlier work indicated that HIV-1 downregulates lymphocyte mRNA translation by suppressing the activity of eIF4E, in a mechanism that is dependent on the accessory protein Vpr (36). Similarly to Se, HIV-1 Vpr promotes the dephosphorylation of 4EBP1 (36), while our data indicate that Gag is able to exclude this active/hypophosphorylated form of 4EBP1 from its 5′ cap association (Fig. 3B) as a potential method to fine-tune gene expression. Regardless of the global reduction in translation, HIV-1 protein synthesis is maintained, as a consequence of a unique composition of the viral mRNP that maintains its association with CBC (36).

Selenium supplementation has been associated with a beneficial role in HIV-1-infected individuals (37), which appears in contrast to the ability of HIV-1 to block protein synthesis imposed by Se. Treatment with increasing concentrations of Se has no effect on cellular Gag, Nef, and Tat synthesis (Fig. 4B), and yet we observed a detrimental effect on the infectivity of released virions (Fig. 4A). Instead, Se affects Gag maturation in purified virus particles, as a decrease in p24^CA^ levels is associated with a concomitant increase in levels of full-length Gag and p160^Gag-Pol^ (Fig. 4B and C), similarly to incubation with the protease inhibitor lopinavir (26). Although HIV-1 is able to counteract the Se-induced block of protein synthesis, our data demonstrate that Se has an important downstream effect on the maturation of released virus particles and may be an important mechanism by which Se imparts a beneficial role in HIV-1 disease progression. Indeed, selenium supplementation intervention trials led to decreased viral loads (10) with clear improvements in survival and reduced morbidity (9, 11–13). Se treatment has been shown to be effective in cancer prevention (38) and selectively toxic toward malignant cells (39–41), in part because Se generates ROS in different cancer cell types (41, 42). Nevertheless, ROS accumulation was not responsible for the reduced infectivity of HIV-1 in the presence of Se, as pretreatment with a superoxidase dismutase (SOD) mimetic (MnTMPyP) does not restore infectivity compared to untreated levels (see Fig. S3 in the supplemental material).

A wide range of cancers specifically associated with HIV-1 infection and immunosuppression is recognized as a major complication of HIV-1/AIDS. Selenium-containing chemotherapeutic agents appear to be promising avenues in the battle against cancer, taking into consideration that Se-induced SGs promote cell death instead of cell survival in response to stress (43). While there are natural sources of selenium from the food, the beneficial effects of selenium supplementation are of clinical interest in the treatment of cancer, heart disease, and immune-related disorders (44). Thus, selenium supplementation could be extremely helpful for the simultaneous treatment of viral infection and tumor progression.

In this work, we describe how HIV-1 Gag possesses yet another role in countering antiviral stress responses. Blocking Se-induced SG assembly via interference with a component of the cap-binding complex is a distinct mechanism and reinforces the notion that SGs are deleterious to HIV-1 replication. Gag’s role in altering the affinity of hypophosphorylated 4EBP1 for eIF4E is a novel countermeasure to elicit an SG blockade for an RNA virus.

## MATERIALS AND METHODS

### Cell culture and transfection conditions.

Human osteosarcoma-derived (U2OS) cells (45) and U2OS GFP-G3BP1-expressing cells (46) were a kind gift from Paul Anderson (Harvard Medical School). HeLa and TZM-bl cells were obtained from the NIH AIDS Reference and Reagent Program. All cells were cultured in Dulbecco’s modified Eagle’s medium (DMEM; Life Technologies) containing 10% fetal bovine serum (FBS) (HyClone) and 1% penicillin-streptomycin (pen/strep) (Life Technologies). Se treatment was highly toxic to HeLa cells (data not shown); therefore, we used U2OS cells, as described previously (14). Cells were transfected using JetPrime (PolyPlus transfections), according to the manufacturer’s instructions.

### HIV-1-infected-patient-derived cells.

PBMCs from an HIV-1-infected patient (healthy untreated progressor) from the Royal Victoria Hospital, Montréal, Québec, Canada, were obtained by leukapheresis and subjected to density-gradient centrifugation using Ficoll (Wisent). These were cultured in RPMI 1640 medium (Life Technologies) containing 10% FBS, 1% pen/strep, and 2 mM l-glutamine (Life Technologies). The patient was a 38-year-old HIV-positive (HIV^+^) untreated male with a viral load of 9,000 copies/ml. All subjects provided informed consent for participating in this study, and human research and ethics committees from the participating study site approved this study.

### Plasmids.

pNL4-3 was used to transfect cells with an infectious proviral HIV-1 molecular clone. pGag-EGFP (47) was obtained from the NIH AIDS Reference and Reagent Program (ARRP). Additionally, this plasmid was used as a template for cloning Gag into pCI-Neo-Flag between XhoI and NotI (pFlag-Gag).

### Drug treatment.

Cells were pretreated with 10 µM MnTMPyP (Enzo Life Sciences) for 1 h to determine the effect of ROS. Cells were subsequently treated with 500 µM sodium Ars (NaAsO_2_; Sigma), 1 mM sodium Se (Na_2_O_3_Se; Sigma), or 250 nM Torin, a kind gift from Nahum Sonenberg, for 45 min, 2 h, or 1 h, respectively, unless stated otherwise.

### Antibodies.

Mouse anti-p24 (NIH ARRP) was used for indirect immunofluorescence microscopy at a dilution of 1:400 and for Western blotting at a dilution of 1:10,000; mouse anti-Tat (NIH ARRP) was used for Western blotting at a dilution of 1:1,000; rabbit anti-Nef (NIH ARRP) was used for Western blotting at a dilution of 1:1,000; mouse anti-biotin (Sigma) was used for Western blotting at a dilution of 1:1,000; rabbit anti-G3BP1 (Santa Cruz Biotechnology) was used for indirect immunofluorescence microscopy at a dilution of 1:1,000 and for Western blotting at a dilution of 1:10,000; goat anti-TIAR (Santa Cruz Biotechnology) was used for indirect immunofluorescence microscopy at a dilution of 1:500; rabbit anti-mTOR, rabbit anti-S6K, rabbit anti-4EBP1, mouse anti-eIF2α, rabbit anti-phospho-mTOR (Ser2448), rabbit anti-phospho-S6K (Thr389), rabbit anti-phospho-4EBP1 (Th37/46), and rabbit anti-phospho-eIF2α (S51) antibodies (Abs) were from Cell Signaling Technology and were all used for Western blotting at a dilution of 1:1,000; goat anti-eIF3 (Abcam) was used for Western blotting at a dilution of 1:1,000; rabbit anti-eIF4E (Abcam) was used for Western blotting at a dilution of 1:1,000; mouse anti-actin (Abcam) was used for Western blotting at a dilution of 1:10,000; and mouse anti-GAPDH (anti-glyceraldehyde-3-phosphate dehydrogenase) (Abcam) was used for Western blotting at a dilution of 1:5,000. Horseradish peroxidase-conjugated secondary antibodies were from Rockland Immunochemicals and used at a dilution of 1:5,000, while Alexa Fluor secondary antibodies were from Life Technologies and used at a dilution of 1:500.

### Western blot analysis.

Cells were lysed in NP-40 lysis buffer (50 mM Tris [pH 7.4], 150 mM NaCl, 0.5 mM EDTA, 0.5% NP-40). Equal amounts of protein were separated by SDS-PAGE and transferred to a nitrocellulose membrane (Bio-Rad). Membranes were probed with the indicated primary and appropriate horseradish peroxidase-conjugated secondary antibodies. Proteins were detected using Western Lightning Plus-ECL (PerkinElmer). For quantitation, the pixel intensity for each band was determined using the ImageJ program (NIH) and then normalized to the indicated control.

### Infectivity assay.

pNL4-3-transfected U2OS cells were treated with increasing concentrations of Se. Virions were collected by ultracentrifugation, and equal volumes were used to infect TZM-bl HeLa cells. Infected cells were lysed, and luciferase activity was measured using the luciferase assay system (Promega).

### Immunofluorescence (IF) and imaging analysis.

Cells were fixed in 4% paraformaldehyde and permeabilized with 0.2% Triton X-100. Primary antibodies were applied followed by incubation in appropriate secondary antibody. Stained cells were mounted in ProLong Gold Antifade Reagent with DAPI (4′,6-diamidino-2-phenylindole; Life Technologies). Laser scanning confocal microscopy was performed using a Leica DM16000B microscope equipped with a WaveFX spinning disk confocal head (Quorum Technologies), and images were acquired with a Hamamatsu ImagEM electron microscopy (EM) charged-coupled-device (CCD) camera. Imaging analyses were performed using Imaris V. 8.1.2 software (Bitplane, Inc.). The observed phenotypes were representative of *n* = 150 cells per condition in each experiment, and SGs were defined as G3BP1 foci (greater than 0.4 µm in diameter [14]).

### *In situ* protein-protein interaction assay (DuoLink).

Unmodified cells were processed for *in situ* proximity ligand assay (PLA) using a DuoLink II *in situ* kit (Olink). The primary antibodies used were mouse anti-p24 and rabbit anti-eIF4E, which were detected using DuoLink II Detection Reagent Red, Duolink II PLA probe anti-Mouse Minus, and DuoLink II PLA probe anti-Rabbit Plus. Imaging was performed as described above. The Spots Tool in Imaris V. 8.1.2 software (Bitplane, Inc.) was used to quantify the number of spots per cell.

### Click-IT and *de novo* Gag synthesis.

To examine the changes in *de novo* synthesis of Gag upon Se treatment, the medium was replaced with methionine-free media containing Click-IT AHA (l-azidohomoalanine) (Life Technologies) (50 µM). Cells were lysed with 1% SDS, and the Click-IT reaction was carried out on 50 µg of protein lysate using a Click-IT Protein Reaction buffer kit (Life Technologies) and biotin-alkyne (40 µM). The labeled material was precleared with normal rabbit serum and 25 µl of a 50:50 slurry of protein G-Sepharose (Thermo Scientific) and incubated with anti-p24 antibody and a 50:50 slurry of protein G-Sepharose.

### Immunoprecipitation and m^7^GTP-pulldown assays.

Due to the low transfection efficiency of U2OS cells, at 48 h posttransfection with GFP or Gag-GFP, these cells were trypsinized and subjected to fluorescence-activated cell sorter (FACS) analysis on the basis of GFP expression twice. Sixty-seven percent of the cell population used in the experiment was GFP positive. After incubation with either Se or Tor, cells were solubilized with NP-40 lysis buffer. Protein lysate was incubated with prewashed anti-GFP monoclonal antibody (MAb) magnetic beads (MLB) or immobilized γ-aminophenyl-m^7^GTP agarose (Jena Bioscience) overnight. Beads were washed before being eluted with 2× Laemmli sample buffer.

## SUPPLEMENTAL MATERIAL

Figure S1  Time course of Se-induced SG assembly. U2OS cells were transfected with pNL4-3 and fixed at the indicated time post-Se stress. Cells were stained for TIAR (cyan), G3BP1 (red), and Gag (green). Scale bars are 10 µm. Images shown are representative of the results of 3 independent experiments. Download Figure S1, TIF file, 2 MB

Figure S2  Characterization of Se-induced SGs. U2OS cells were transfected with pNL4-3 and stressed with Se for 2 h. (A) Cells were stained for DAPI (blue), G3BP1 (green), TIAR (yellow), and Gag (red). (B) pNL4-3-transfected cells were treated with Ars or Se and stained for eIF3 (blue), G3BP1 (green), and Gag (red). White arrowheads indicate non-HIV-1-expressing cells, while red arrowheads show those cells that were expressing Gag. Green arrowheads indicate SGs. Scale bars are 10 µm. Images shown are representative of the results of 3 independent experiments. (C) U2OS GFP-G3BP-expressing cells were pretreated with the ROS scavenger MnTMPyP (10 µM) for 1 h before treatment with Se without or with MnTMPyP. Cells were stained for DAPI (blue) and TIAR (cyan). Download Figure S2, TIF file, 2 MB

Figure S3  HIV-1-infected Jurkat T cells lack Se-induced SGs. Jurkat T cells were infected with NL4-3 in the presence of 6 µg/ml Polybrene and centrifuged at 1,800 rpm for 90 min. After centrifugation, cells were washed and incubated in fresh RPMI 1640 medium supplemented with 10% FBS. At 12 days postinfection, cells were treated with Se for 2 h before being washed and fixed. Cells were stained for DAPI (blue), G3BP1 (red), TIAR (cyan), and Gag (green). White arrowheads indicate non-HIV-1-expressing cells, while red arrowheads show those cells that were expressing Gag. Green arrowheads indicate SGs with colocalization of G3BP1 and TIAR. Scale bars are 7 µm. Download Figure S3, TIF file, 1.1 MB

Figure S4  PLA of Gag and eIF4E in pNL4-3-trasnfected HeLa cells. (A) HeLa cells transfected with pcDNA3.1 or pNL4-3 were fixed, permeabilized, and incubated with mouse anti-p24 and rabbit anti-eIF4E. Each red dot corresponds to a single event of interaction between Gag and eIF4E. Nuclei were stained with DAPI (blue). Images shown are representative of 70 cells analyzed from 2 independent experiments. The graph indicates the number of dots per cell. Asterisks represent statistically significant differences between pcDNA3.1 and pNL4-3 (Student’s *t* test; *P* < 0.001). (B) Cell lysates from U2OS GFP or Gag-GFP cells were collected and subjected to m^7^GTP agarose pulldown in the absence or presence of RNase A. Cap-associated proteins were processed for Western blotting and probed for p24 and eIF4E. Download Figure S4, TIF file, 0.7 MB

Figure S5  ROS production by Se does not affect released HIV-1 particles. U2OS cells were transfected with pNL4-3 and pretreated without or with the ROS scavenger MnTMPyP for 1 h before being stressed with increasing concentrations of Se for 2 h. Virus-containing supernatants were filtered, collected by ultracentrifugation, and subjected to Western blotting using an anti-p24 antibody. Download Figure S5, TIF file, 0.7 MB
