# Supplementary figures and images for: HIV-1 Gag Blocks Selenite-Induced Stress Granule Assembly by Altering the mRNA Cap-Binding Complex
Source: mBio. 2016 Mar 29;7(2):e00329-16. doi: 10.1128/mBio.00329-16 (PMC4817256; doi:10.1128/mBio.00329-16)

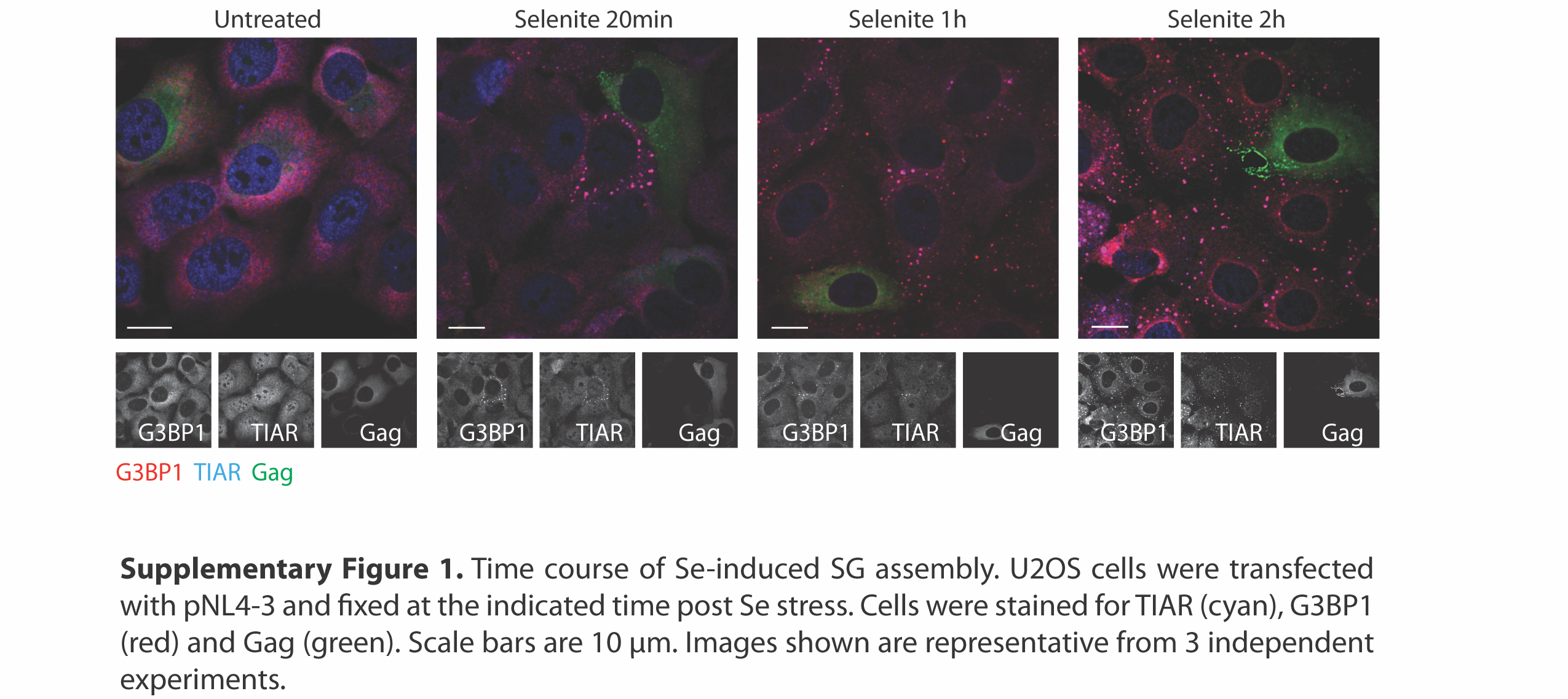

Supplement: Figure S1 — Time course of Se-induced SG assembly. U2OS cells were transfected with pNL4-3 and fixed at the indicated time post-Se stress. Cells were stained for TIAR (cyan), G3BP1 (red), and Gag (green). Scale bars are 10 µm. Images shown are representative of the results of 3 independent experiments. Download [file mbo004142754sf1.tif]

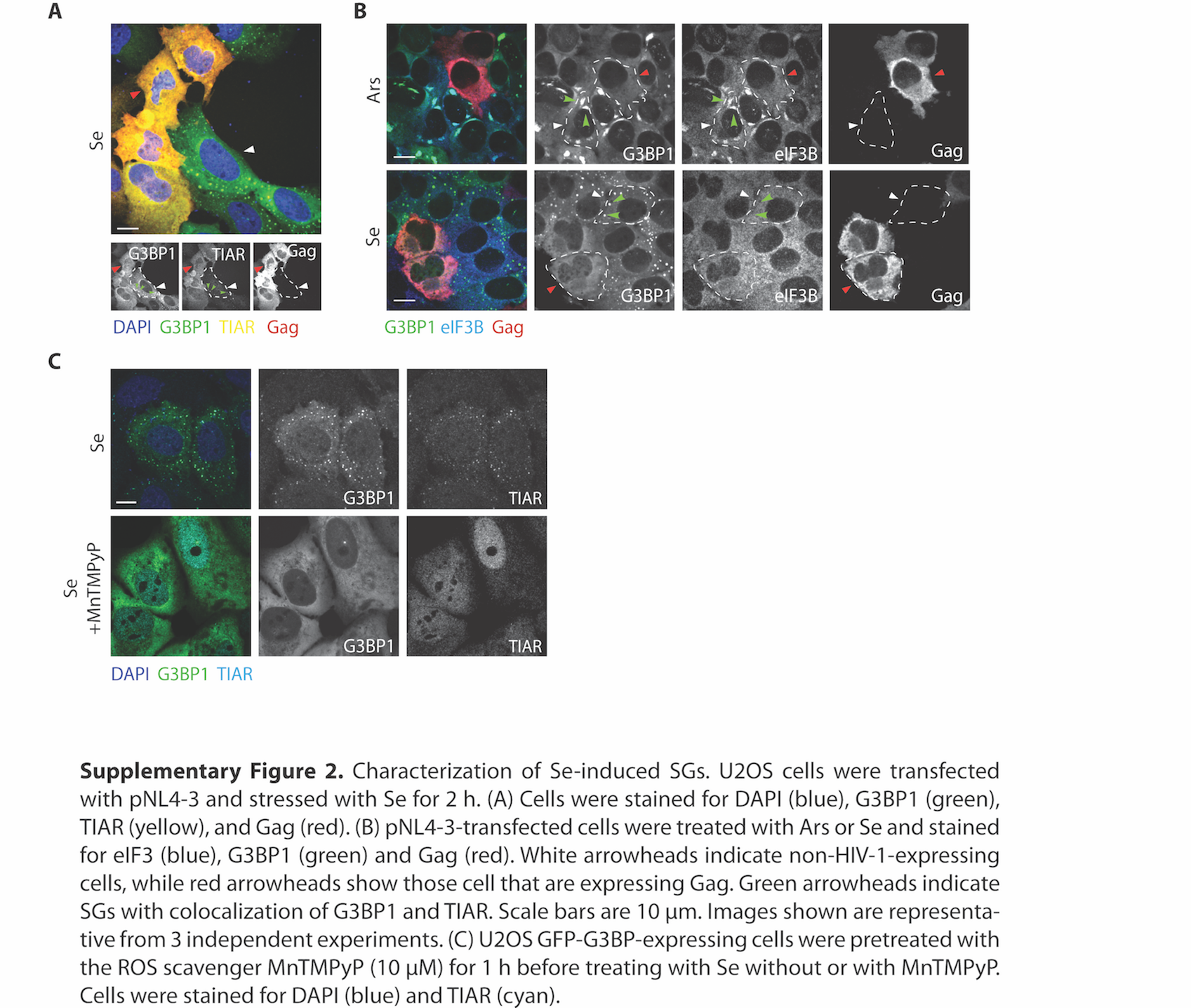

Supplement: Figure S2 — Characterization of Se-induced SGs. U2OS cells were transfected with pNL4-3 and stressed with Se for 2 h. (A) Cells were stained for DAPI (blue), G3BP1 (green), TIAR (yellow), and Gag (red). (B) pNL4-3-transfected cells were treated with Ars or Se and stained for eIF3 (blue), G3BP1 (green), and Gag (red). White arrowheads indicate non-HIV-1-expressing cells, while red arrowheads show those cells that were expressing Gag. Green arrowheads indicate SGs. Scale bars are 10 µm. Images shown are representative of the results of 3 independent experiments. (C) U2OS GFP-G3BP-expressing cells were pretreated with the ROS scavenger MnTMPyP (10 µM) for 1 h before treatment with Se without or with MnTMPyP. Cells were stained for DAPI (blue) and TIAR (cyan). Download [file mbo004142754sf2.tif]

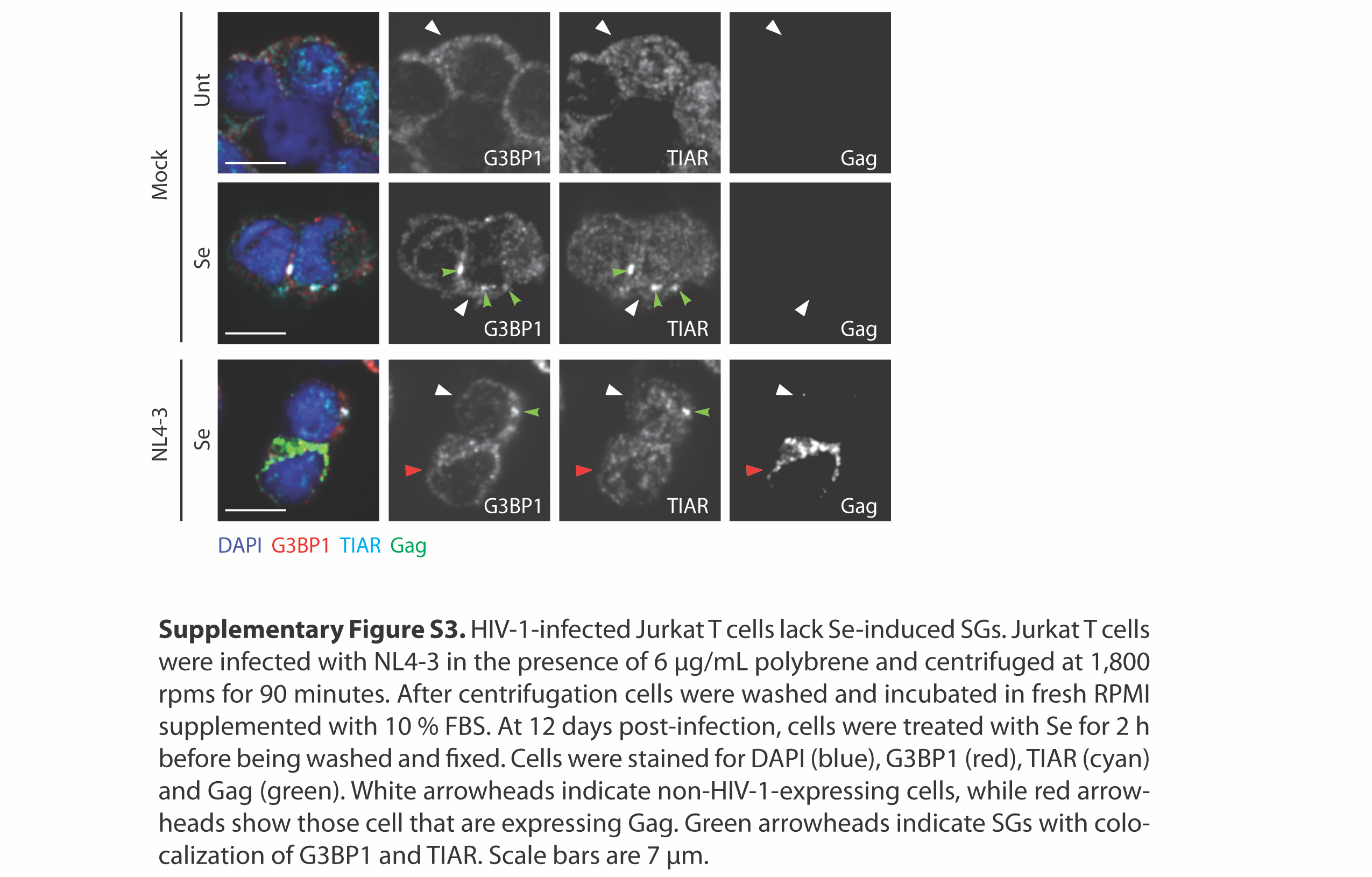

Supplement: Figure S3 — HIV-1-infected Jurkat T cells lack Se-induced SGs. Jurkat T cells were infected with NL4-3 in the presence of 6 µg/ml Polybrene and centrifuged at 1,800 rpm for 90 min. After centrifugation, cells were washed and incubated in fresh RPMI 1640 medium supplemented with 10% FBS. At 12 days postinfection, cells were treated with Se for 2 h before being washed and fixed. Cells were stained for DAPI (blue), G3BP1 (red), TIAR (cyan), and Gag (green). White arrowheads indicate non-HIV-1-expressing cells, while red arrowheads show those cells that were expressing Gag. Green arrowheads indicate SGs with colocalization of G3BP1 and TIAR. Scale bars are 7 µm. Download [file mbo004142754sf3.tif]

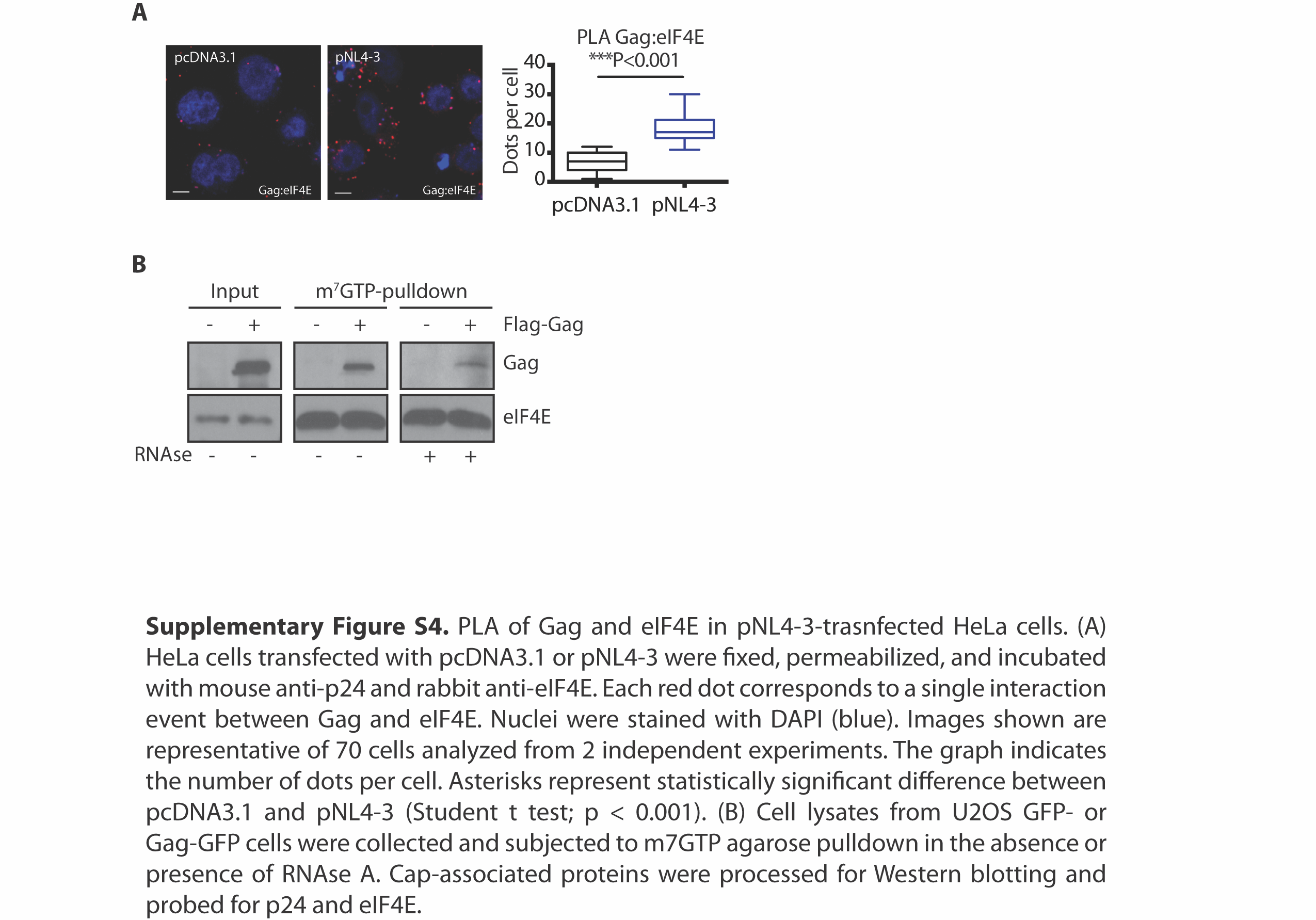

Supplement: Figure S4 — PLA of Gag and eIF4E in pNL4-3-trasnfected HeLa cells. (A) HeLa cells transfected with pcDNA3.1 or pNL4-3 were fixed, permeabilized, and incubated with mouse anti-p24 and rabbit anti-eIF4E. Each red dot corresponds to a single event of interaction between Gag and eIF4E. Nuclei were stained with DAPI (blue). Images shown are representative of 70 cells analyzed from 2 independent experiments. The graph indicates the number of dots per cell. Asterisks represent statistically significant differences between pcDNA3.1 and pNL4-3 (Student’s t test; P < 0.001). (B) Cell lysates from U2OS GFP or Gag-GFP cells were collected and subjected to m7GTP agarose pulldown in the absence or presence of RNase A. Cap-associated proteins were processed for Western blotting and probed for p24 and eIF4E. Download [file mbo004142754sf4.tif]

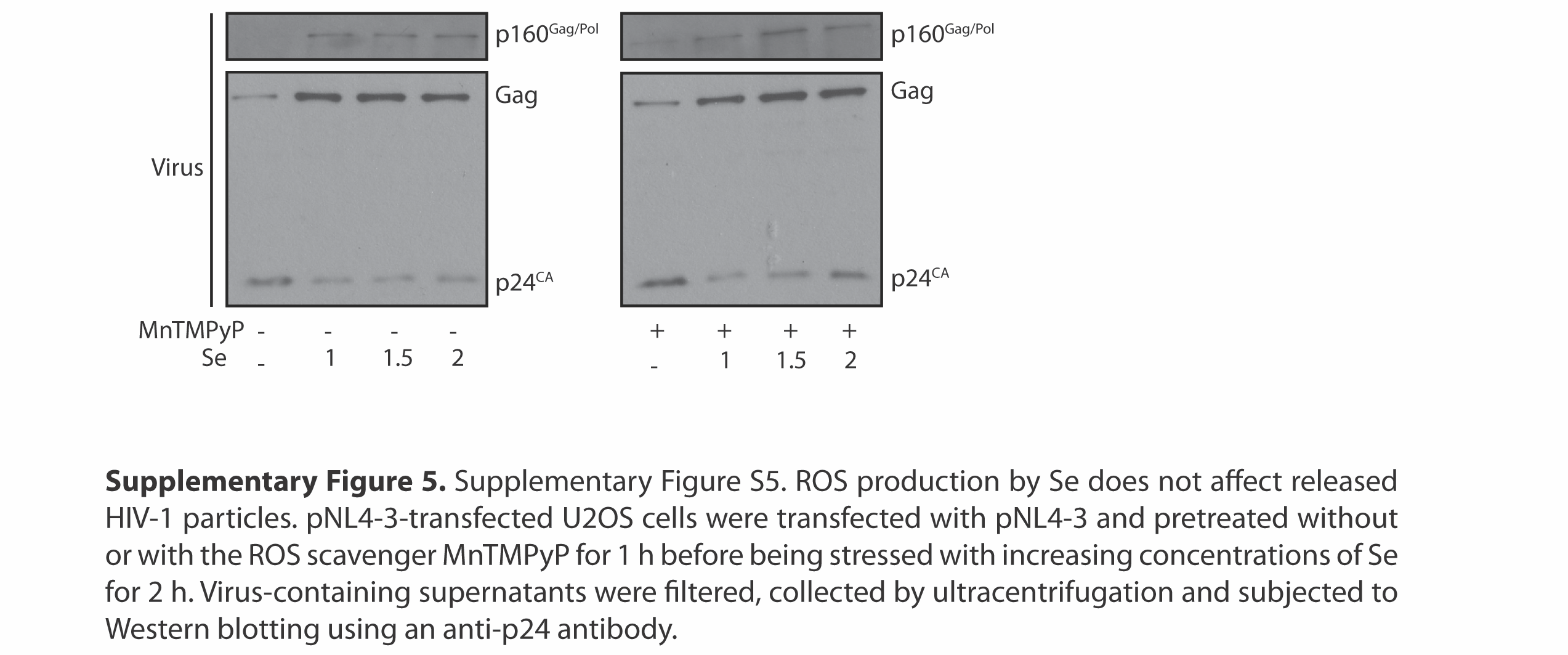

Supplement: Figure S5 — ROS production by Se does not affect released HIV-1 particles. U2OS cells were transfected with pNL4-3 and pretreated without or with the ROS scavenger MnTMPyP for 1 h before being stressed with increasing concentrations of Se for 2 h. Virus-containing supernatants were filtered, collected by ultracentrifugation, and subjected to Western blotting using an anti-p24 antibody. Download [file mbo004142754sf5.tif]
